# Epithelioid Inflammatory Myofibroblastic Sarcoma: A Report of a Rare Case

**DOI:** 10.7759/cureus.68184

**Published:** 2024-08-30

**Authors:** Varun Ronanki, Vaddatti Tejeswini, Inuganti Venkata Renuka, Shaik Raheema, Bakkamanthala S K Kanth

**Affiliations:** 1 Pathology, NRI Medical College, Guntur, IND

**Keywords:** rare, lung, anaplastic lymphoma kinase, myofibroblastic, sarcoma

## Abstract

Epithelioid inflammatory myofibroblastic sarcoma (EIMS), a variant of inflammatory myofibroblastic tumor (IMT), is a rare malignant tumor commonly associated with anaplastic lymphoma kinase (ALK) gene fusions and is aggressive in nature with local recurrence. Here, we report a case of a 23-year-old female who presented with a cough and, upon investigations, was found to have a mass in the left upper lobe of the lung detected by chest computed tomography (CT). Biopsy revealed EIMS with ALK and desmin protein expression. The patient underwent a lobectomy via video-assisted thoracoscopic surgery (VATS). The postoperative period was uneventful.

## Introduction

Epithelioid inflammatory myofibroblastic sarcoma (EIMS) is a rare subtype of inflammatory myofibroblastic tumor (IMT), characterized by epithelioid-to-round cell morphology and prominent inflammatory infiltrate [1]. It tends to occur predominantly in children and young adults, with a higher prevalence in males compared to females [2], unlike IMT, which is slightly more common in females. EIMS is often associated with RANBP2-ALK or RRBP1-ALK gene rearrangements [3]. EIMS primarily manifests in the intra-abdominal region, while involvement of the liver and lung is rare [3,4]. One of the key distinctions between EIMS and IMT lies in their clinical behavior and aggressiveness. EIMS is known to be more aggressive, with recurrence and metastasis rates exceeding 80% and 25%, respectively [5]. Clinically, EIMS typically presents as a rapidly growing intra-abdominal mass or a pleural nodule, often accompanied by symptoms such as abdominal pain, ascites, or pleural effusion [6]. This case is presented in view of its rare presentation and to increase awareness for diagnosis.

## Case presentation

**Figure 1:**
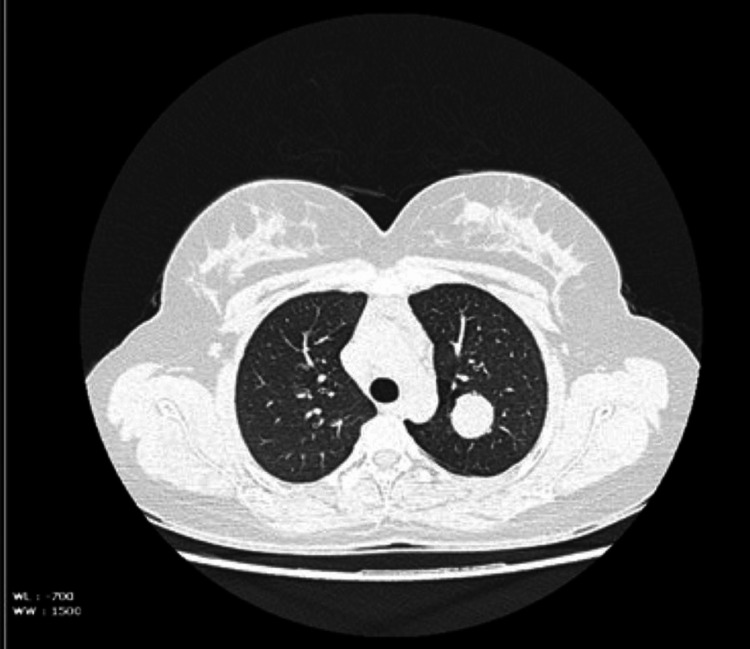
CECT lung shows an intraparenchymal soft tissue density lesion measuring 2.5 x 2.2 x 2.3 cm noted in the apicoposterior segment of the left upper lobe, suggestive of a solitary pulmonary nodule. CECT: contrast enhanced computed tomography

This is a case of a 23-year-old female presented with a history of dry cough for one week. The routine blood investigations were not significant. Her chest X-ray revealed a nodular homogenous opacity measuring 2.8 × 2.4 cm in the left upper zone. Subsequent CT and contrast-enhanced computed tomography (CECT) imaging of the chest showed a round soft tissue density lesion with lobulated margins of 2.5 x 2.2 x 2.3 cm in the left upper lobe, as in Figure 1.

**Figure 2:**
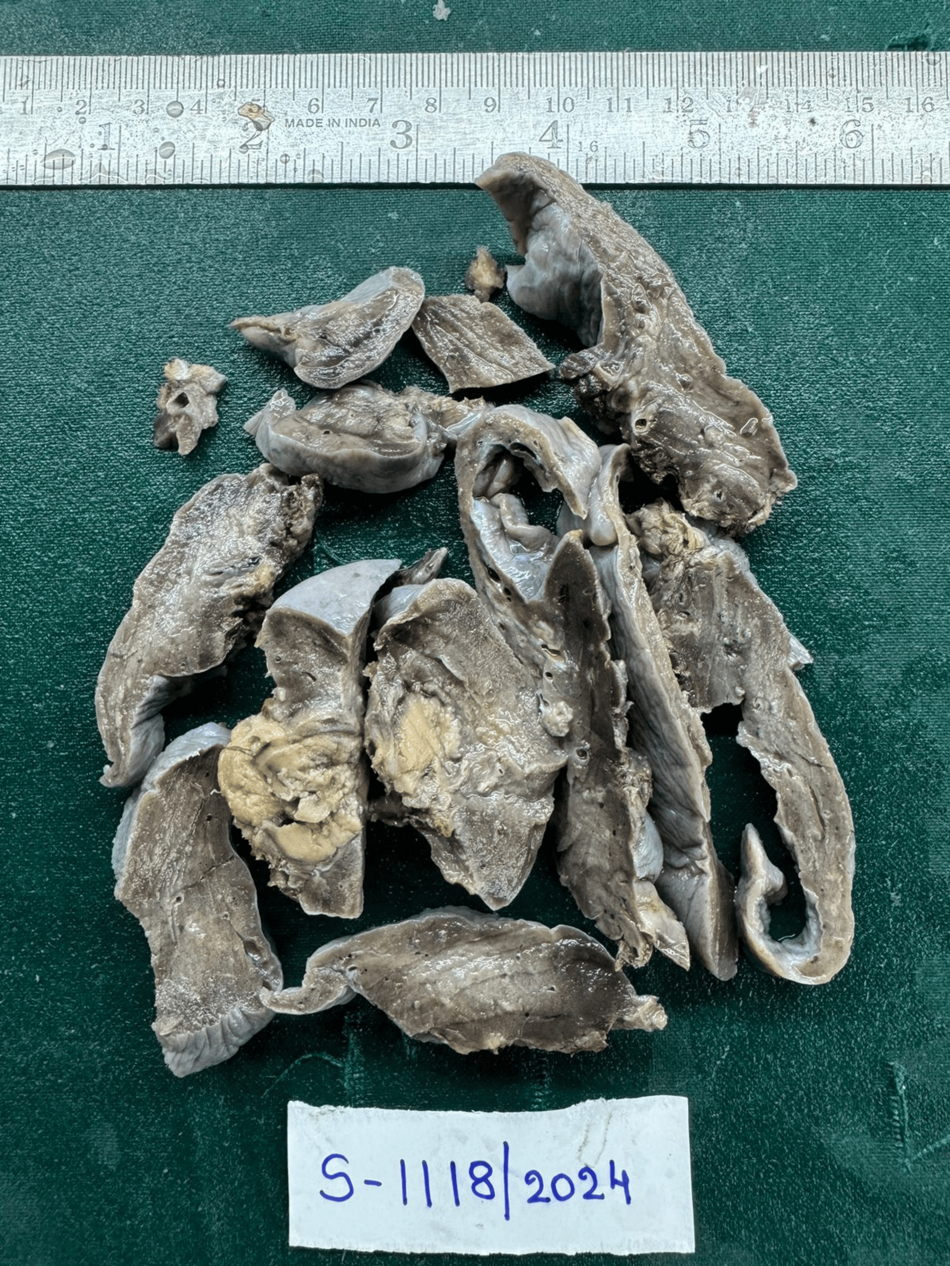
Cut section of mass showing irregular grey, yellow lesion of 3 x 2.5 cm.

These findings collectively suggested a "solitary pulmonary nodule." Tru-cut biopsy revealed the features of EIMS, which were confirmed by immunohistochemistry (IHC) markers showing negativity for smooth muscle actin (SMA) and positivity for desmin and anaplastic lymphoma kinase (ALK). A left lobectomy was conducted using video-assisted thoracoscopic surgery (VATS), which unveiled the tumor in two distinct pieces. The two segments were of sizes 11 x 7 x 1 cm and 6 x 4 x 3 cm, respectively, as shown in Figure 2.

**Figure 3:**
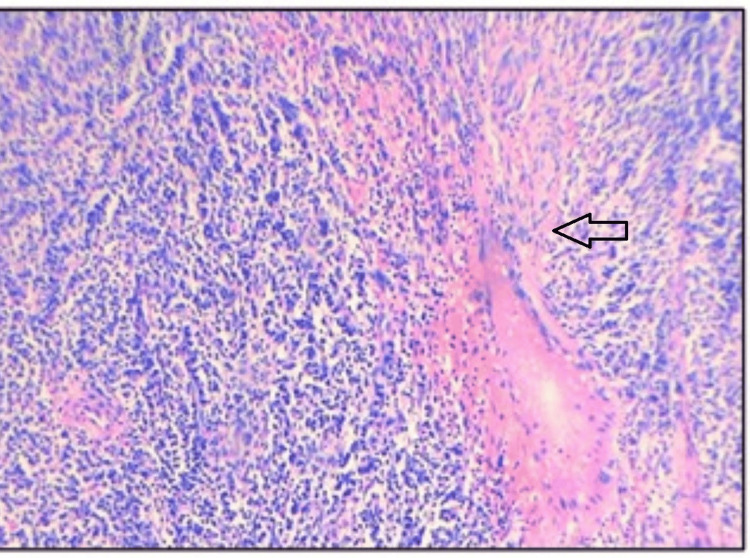
Microscopic photographs of EIMS showing (H&E: 100X) sections of lesion composed of oval to spindle-shaped neoplastic cells in fascicles separated by thick fibrous septa. EIMS: epitheloid inflammatory myofibroblastic sarcoma; H&E: hematoxylin & eosin (100X magnification)

**Figure 4:**
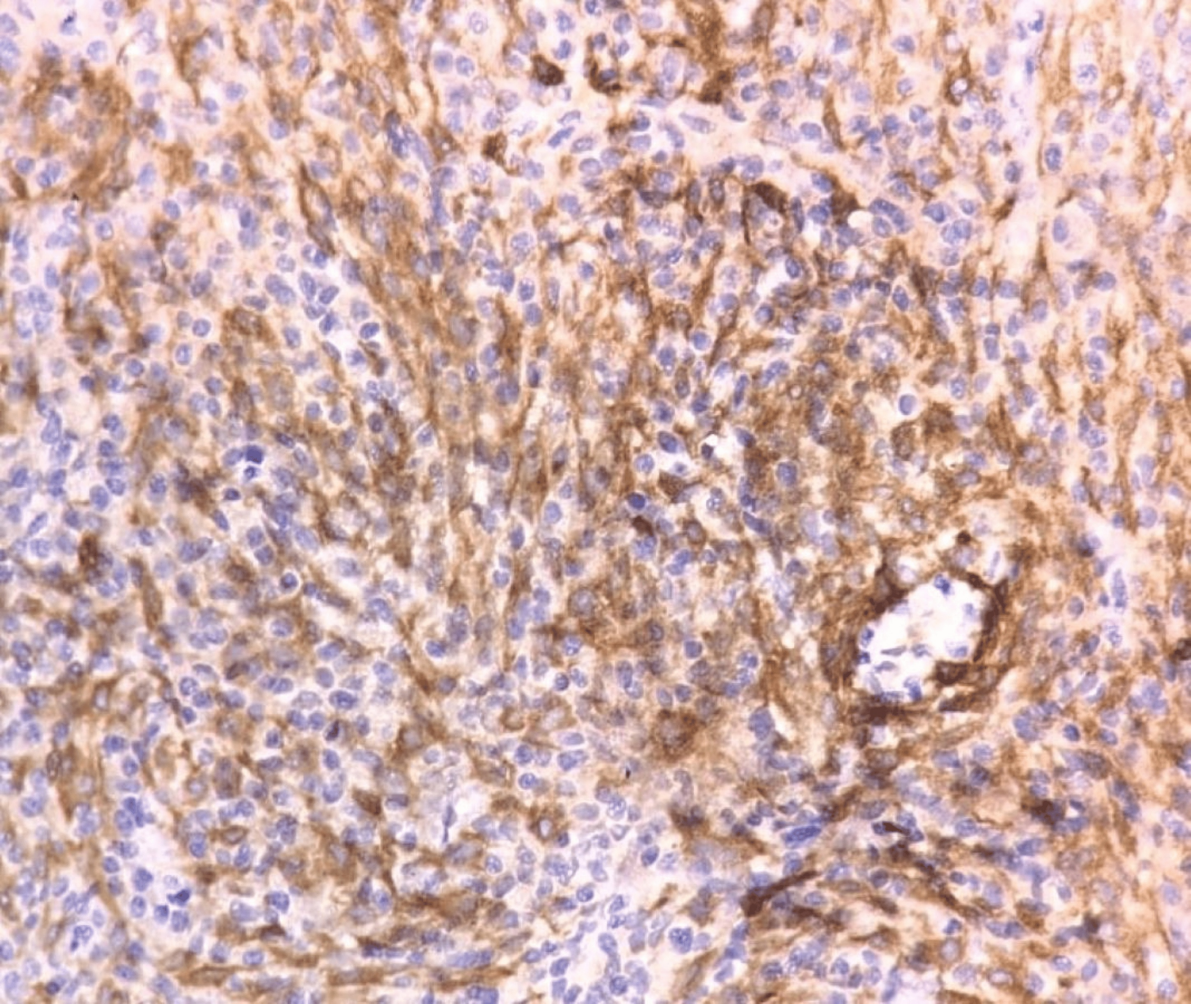
IHC showing ALK positivity in tumor cells. IHC: immunohistochemistry; ALK: anaplastic lymphoma kinase

**Figure 5:**
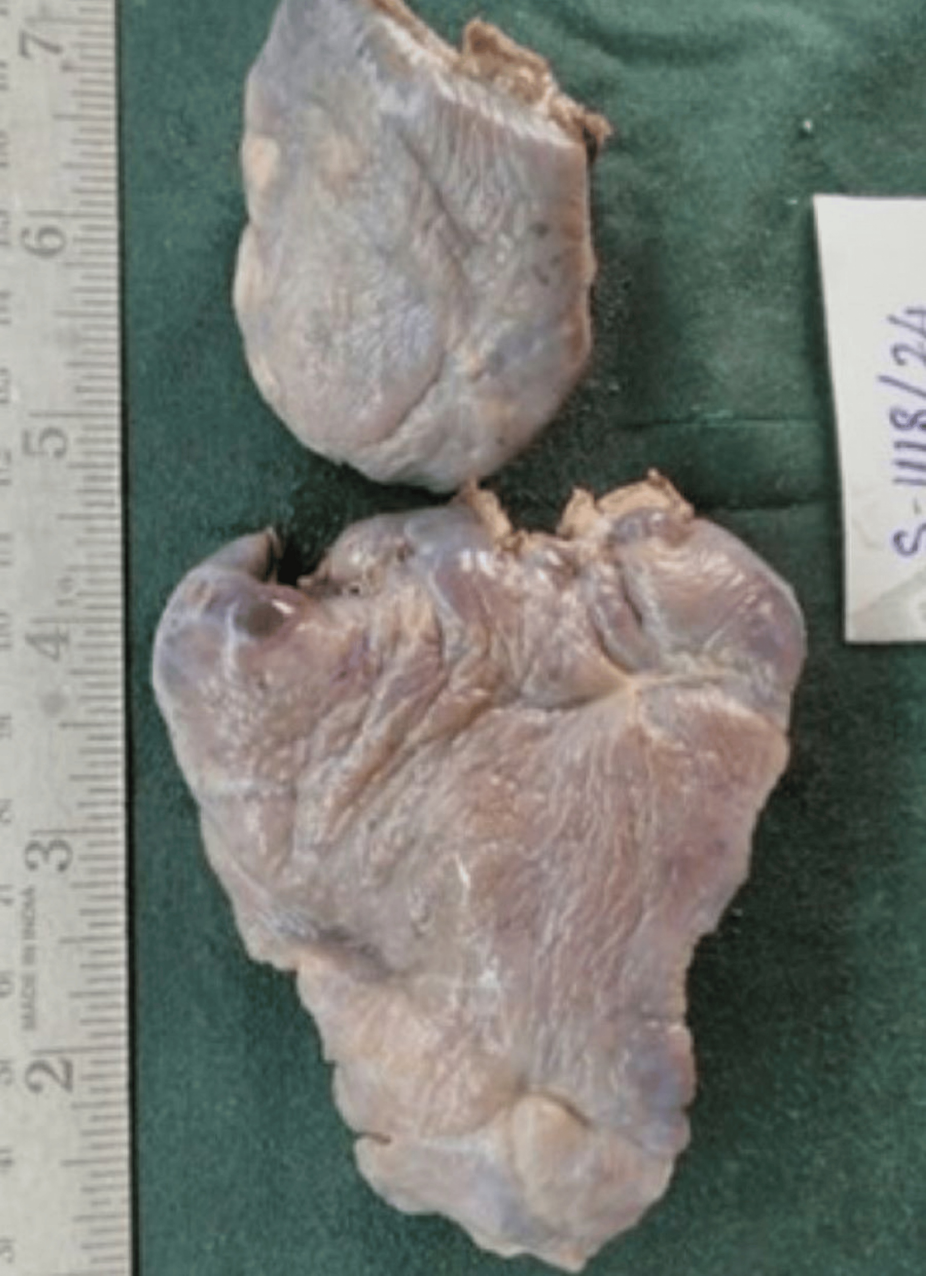
Excised lobectomy specimen of left upper lobe of lung grossly in two pieces, the largest measuring 11 x 7 x 1 cm and the smallest measuring 6 x 4 x 3 cm. External surface-smooth.

**Figure 6:**
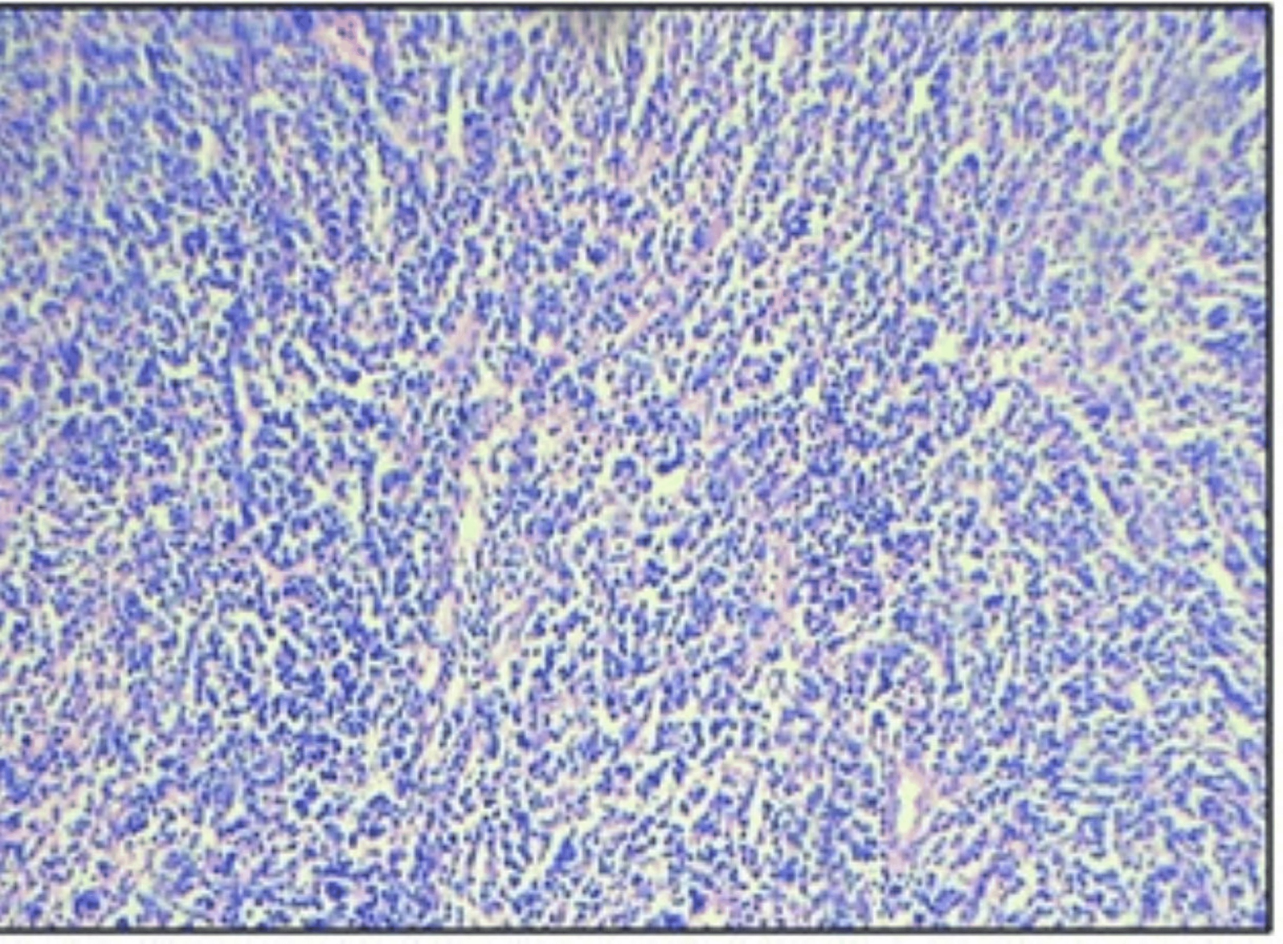
Microscopic photographs of EIMS showing (H&E: 100X) plump round epithelioid cells with an oval blunt nucleus and vesicular chromatin. EIMS: epitheloid inflammatory myofibroblastic sarcoma; H&E: hematoxylin & eosin (100x magnification)

**Figure 7:**
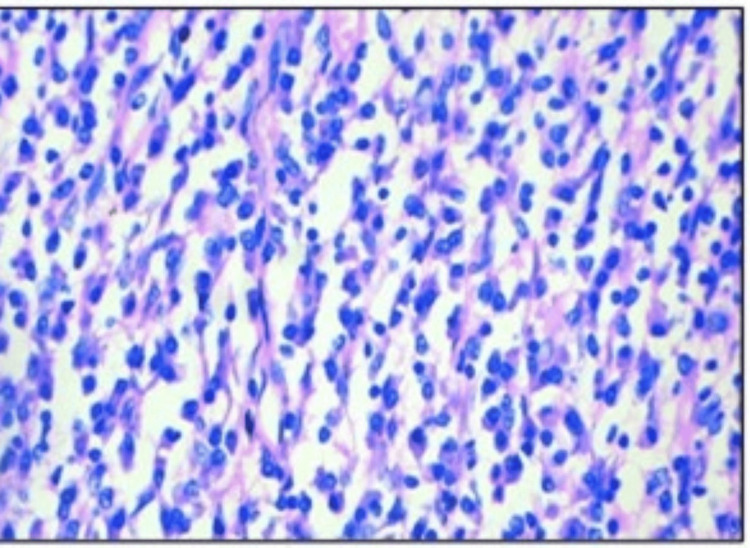
Microscopic picture of EIMS H&E 400x oval to spindle-shaped cells with plump blunt enlarged nucleus, vesicular chromatin, prominent nucleoli in some, and moderate eosinophilic cytoplasm. EIMS: epitheloid inflammatory myofibroblastic sarcoma; H&E: hematoxylin & eosin (400X magnification)

**Figure 8:**
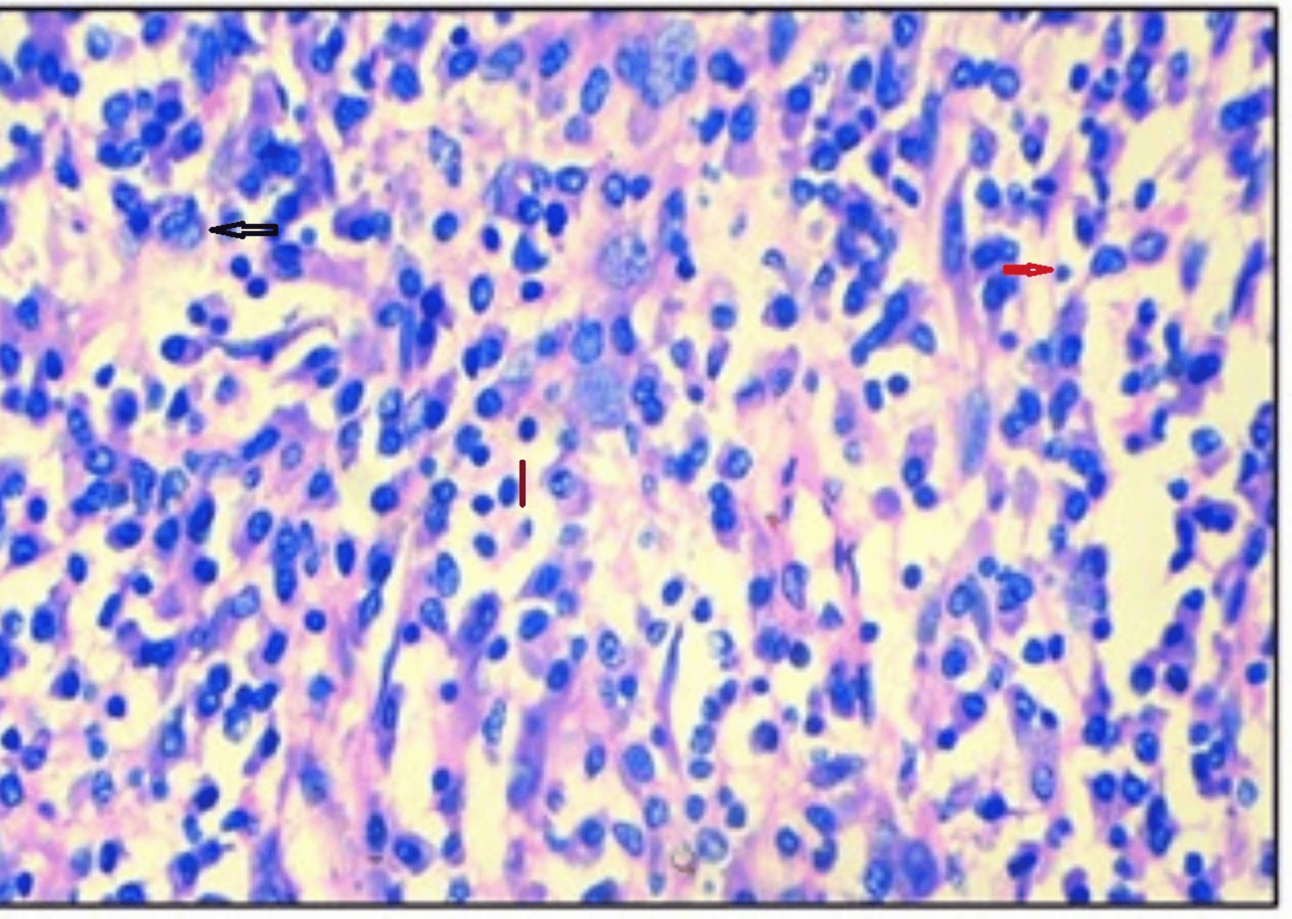
Microscopic picture of EIMS (H&E: 400X) epithelioid tumor cells admixed with dense chronic inflammatory infiltrate with lymphocytes and plasma cells. Black arrow plump nucleus with vesicular chromatin and prominent nucleoli; red arrow shows plasma cell with eccentrically placed nucleus; orange arrow shows lymphocyte. EIMS: epitheloid inflammatory myofibroblastic sarcoma; H&E: hematoxylin & eosin (400x magnification)

Histopathological examination revealed hypocellular and hypercellular regions. The tumor shows fascicles of plump and spindle-shaped neoplastic cells with plump vesicular nucleus and abundant eosinophilic cytoplasm, indicative of an epithelioid configuration. An additional spindle cell component was noted, partially obscured by dense lymphoplasmacytic infiltrates. Occasional foreign body-type giant cells were seen. Notably, there were no areas of necrosis seen, and mitotic activity was minimal, along with normal lung tissue. Figures 3, 4, 5, 6 depict the features. Immunohistochemistry revealed ALK positivity as shown in Figures 7, 8.

Postoperative follow-up after three months was uneventful.

## Discussion

Marino Enriquez et al. first identified EIMS in 2011 [7]. A total of 46 cases were published on EIMS as of the literature dated July 30, 2020.

EIMS is characterized by large epithelioid cells with round nuclei, prominent nucleoli, and vesicular chromatin in a background of myxoid stroma and neutrophil-rich inflammatory infiltrate that show mitosis and necrosis [2]. EIMS is often associated with gene rearrangements involving the anaplastic lymphoma kinase (ALK) gene, the most common being RANBP2-ALK and RRBP1-ALK. The fusions have been associated with a peculiar nuclear membrane (RANBP2-ALK fusion) or perinuclear accentuated cytoplasmic immunostaining (RRBP1-ALK fusion) for ALK protein [2].

EIMS commonly occurs in the abdomen and pelvis; its occurrence in the lung was initially reported by Fu et al. [6]. The patient described in their report presented with weight loss and fatigue. Subsequent cases of EIMS found in the pleura were documented by Sarmiento et al. and Kozu et al. presenting with dyspnea and pleural effusion [8,9]. The present case presented primarily with only cough. EIMS tends to occur more in children and young adults, more commonly in males [2]. In contrast to our case where we reported a young female diagnosed with EIMS as depicted in Table 1.

**Table 1:** Clinicopathological features of reported cases of epithelioid inflammatory myofibroblastic sarcoma including the current case M: male; F: female; ALK: anaplastic lymphoma kinase; SMA: smooth muscle actin; RANBP2: RAN binding protein 2; CD 30: cluster of differentiation 30; FISH: flourescence in situ hybridization; PCR: polymerase chain reaction; N/A: not applicable

S.N o.	Study	Age (yr)/Sex	Anatomic site	Size (cms)	Clinical presentation	Immunoprofile	Molecular confirmation	Treatment
1.	Fu et al. [6].	21/M	Left lower lobe, lung	10	Weight loss, fatigue	Desmin+, ALK (c), CD30–	*ALK* rearrangement (FISH)	Lobectomy
2.	Sarmiento et al. [8].	71/M	Pleura based, left lower lobe	12.5	Dyspnoea, pleural effusion	N/A	*ALK* rearrangement (FISH)	Lobectomy and adjuvant crizotinib
3.	Kozu et al. [9].	57/M	Pleural cavity/chest wall	N/A	Pleural effusion and dyspnoea	Desmin+, cytokeratin+, ALK+ (c), CD30–	*RANBP2-ALK* fusion (PCR)	Crizotinib
4.	Present study	23/F	Left upper lobe, lung	3	Cough	Desmin+, SMA-, ALK+	N/A	Lobectomy

Diagnosing EIMS based solely on a traditional biopsy presents significant challenges due to its rarity and atypical clinical presentation. Therefore, additional tests for ALK rearrangements, such as fluorescence in situ hybridization (FISH), reverse transcription-polymerase chain reaction (RT-PCR), or next-generation sequencing (NGS), are recommended to definitively diagnose EIMS10 as done in the cases of Fu et al., Sarmiento et al., and Kozu et al. [6,8,9]. However, strong desmin positivity and perinuclear or cytoplasmic ALK positivity have consistently been observed in all reported cases of EIMS [10], which aligns with the present case. Additionally, the negative result for SMA further supports this diagnosis.

The gold standard treatment for EIMS typically involves surgical resection, if necessary supplemented by chemotherapy and/or radiotherapy. A high risk of recurrence is seen in cases of incomplete removal of the tumor. Second-line treatment options may include nonsteroidal anti-inflammatory drugs, high-dose corticosteroids, biological agents, chemotherapy, and radiotherapy. For cases of multifocal, refractory disease, or unresectable tumors, ALK inhibitors such as crizotinib demonstrated efficacy specifically in ALK-rearranged tumors [11]. In our case, the primary treatment approach was the surgical resection of the affected part of the lung via VATS.

Effective management of EIMS is necessary to prevent recurrence and metastasis. The College of American Pathologists (CAP) and the American Joint Committee on Cancer (AJCC) recommend that IMTs be classified using the Pathologic Soft Tissue Stage Classification (pTNM; AJCC 8th Edition). In the report by Fu et al. [6], pelvic and vertebral bone metastases were seen in three months after the surgery, which resulted in mortality. However, in the cases of Sarmiento et al. [8] and Kozu et al. [9], patients were treated with crizotinib. Though in the present case, there was no recurrence in three months, close follow-up is recommended given the aggressive behavior. The present case was of intermediate grade and stage T3.

## Conclusions

EIMS is a rare subtype of inflammatory myofibroblastic tumor (IMT) that typically presents with local metastasis and recurrence. While EIMS most commonly occurs in males and manifests as an intra-abdominal tumor, here we present the rarity of EIMS occurring in the lung of a young female patient who was relatively asymptomatic aside from a cough. Diagnosing EIMS is crucial, and it requires careful differentiation from other tumor types such as inflammatory pseudotumor (IPT), conventional IMT, and soft tissue sarcomas like epithelioid sarcoma or inflammatory liposarcoma. Additionally, it is essential to consider granulomatous diseases like sarcoidosis or granulomatosis with polyangiitis (Wegener's granulomatosis) in the differential diagnosis. Early and accurate diagnosis of EIMS is essential as it can aid in prompt initiation of treatment, which may include surgical resection, chemotherapy, and targeted therapy in cases where ALK gene rearrangement is present. Timely intervention can help prevent recurrences and metastases, ultimately improving patient outcomes.

## References

[1] Li M, Xing R, Huang J, Shi C, Wei C, Wang H (2023). Case report: epithelioid inflammatory myofibroblastic sarcoma treated with an ALK TKI ensartinib. Front Oncol.

[2] Singh P, Nambirajan A, Gaur MK, Raj R, Kumar S, Malik PS, Jain D (2022). Primary pulmonary epithelioid inflammatory myofibroblastic sarcoma: a rare entity and a literature review. J Pathol Transl Med.

[3] Song W, Zhu Y (2021). Clinical characteristics and outcomes of 17 cases of inflammatory myofibroblastic tumor at a University Hospital in China. Oncol Lett.

[4] Gadeyne L, Creytens D, Dekeyser S, Van der Meulen J, Haspeslagh M (2022). Primary cutaneous epithelioid inflammatory myofibroblastic sarcoma harboring RANBP2-ALK fusion: Report of an exceptional case. Am J Dermatopathol.

[5] Giannaki A, Doganis D, Giamarelou P, Konidari A (2021). Epithelioid inflammatory myofibroblastic sarcoma presenting as gastrointestinal bleed: Case report and literature review. JPGN Rep.

[6] Fu X, Jiang J, Tian XY, Li Z (2015). Pulmonary epithelioid inflammatory myofibroblastic sarcoma with multiple bone metastases: case report and review of literature. Diagn Pathol.

[7] Mariño-Enríquez A, Wang WL, Roy A (2011). Epithelioid inflammatory myofibroblastic sarcoma: an aggressive intra-abdominal variant of inflammatory myofibroblastic tumor with nuclear membrane or perinuclear ALK. Am J Surg Pathol.

[8] Sarmiento DE, Clevenger JA, Masters GA, Bauer TL, Nam BT (2015). Epithelioid inflammatory myofibroblastic sarcoma: a case report. J Thorac Dis.

[9] Kozu Y, Isaka M, Ohde Y, Takeuchi K, Nakajima T (2014). Epithelioid inflammatory myofibroblastic sarcoma arising in the pleural cavity. Gen Thorac Cardiovasc Surg.

[10] Dou W, Guan Y, Liu T (2023). Epithelioid inflammatory myofibroblastic sarcoma: a case report and brief literature review. Front Oncol.

[11] George DM, Lakshmanan A, Annapurneswari S, Usharani K (2024). Epithelioid inflammatory myofibroblastic sarcoma - a rare case report. Human Pathology Reports.

